# Neural Components of Reading Revealed by Distributed and Symbolic Computational Models

**DOI:** 10.1162/nol_a_00018

**Published:** 2020-10-01

**Authors:** Ryan Staples, William W. Graves

**Affiliations:** Department of Psychology, Rutgers University, Newark, NJ; Department of Psychology, Rutgers University, Newark, NJ

**Keywords:** language, reading, fMRI, computational modelling, cognitive neuroscience, orthography

## Abstract

Determining how the cognitive components of reading—orthographic, phonological, and semantic representations—are instantiated in the brain has been a long-standing goal of psychology and human cognitive neuroscience. The two most prominent computational models of reading instantiate different cognitive processes, implying different neural processes. Artificial neural network (ANN) models of reading posit nonsymbolic, distributed representations. The dual-route cascaded (DRC) model instead suggests two routes of processing, one representing symbolic rules of spelling–to–sound correspondence, the other representing orthographic and phonological lexicons. These models are not adjudicated by behavioral data and have never before been directly compared in terms of neural plausibility. We used representational similarity analysis to compare the predictions of these models to neural data from participants reading aloud. Both the ANN and DRC model representations corresponded to neural activity. However, the ANN model representations correlated to more reading-relevant areas of cortex. When contributions from the DRC model were statistically controlled, partial correlations revealed that the ANN model accounted for significant variance in the neural data. The opposite analysis, examining the variance explained by the DRC model with contributions from the ANN model factored out, revealed no correspondence to neural activity. Our results suggest that ANNs trained using distributed representations provide a better correspondence between cognitive and neural coding. Additionally, this framework provides a principled approach for comparing computational models of cognitive function to gain insight into neural representations.

## INTRODUCTION

To better understand how the brain carries out a cognitive process, we must have robust approaches to both cognitive models and neural functions. Cognitive models provide a mechanistic explanation of cognitive function, and computational implementations of these models provide explicit and testable predictions of how these processes interact (Forstmann et al., 2011). However, these models alone cannot reveal the neural bases or implementation of the modelled processes. Neuroimaging has separately contributed to localizing where in the brain certain aspects of cognition are processed, but localization alone does not explain cognition. Furthermore, in the absence of model-based constraints, the interpretation of fMRI data is radically underconstrained (Haxby et al., 2014). The union of computational cognitive models and neuroimaging, implemented by recent advances in methodology, allows for the quantitative comparison of specific, model-generated predictions to test their biological plausibility. Here we used this approach to better determine the neural and cognitive basis of reading.

Reading is a particularly promising domain to investigate, given the existence of well-established computational cognitive models and the rich availability of cognitive neuroscience data. Rather than assume the primacy of a particular model, we took a model comparison approach (Popov et al., 2018) to testing for correspondence between two different computational cognitive models optimized to account for reading performance.

One approach to modelling reading uses artificial neural networks (ANNs). ANN models of reading are distributed, nonsymbolic, and based on connectionist principles. They consist of weighted connections, learned via the backpropagation algorithm, between layers of interconnected, neurally inspired units (Plaut et al., 1996; Seidenberg & McClelland, 1989). ANNs map inputs to outputs using some number of “hidden” layers, in which the statistical regularities of the target outputs are related to those of the inputs (Hinton, 1989).

Another prominent model of reading is the symbolic dual-route cascaded (DRC) model (Coltheart et al., 2001). The DRC model also uses weighted connections between nodes for producing phonological output from orthographic input. Unlike ANN models, the DRC model consists of hand-tuned connections among symbolic representations of explicit rules and lexicons.

Both ANN and DRC models have been designed and tested to replicate aspects of human reading, but the results of these simulations and independent evidence for them have so far not adjudicated between the models. Additionally, neither model matches human behavioral data perfectly. For example, an ANN model accurately reproduces the frequency-regularity interaction found in single word reading (Plaut et al., 1996; Seidenberg & McClelland, 1989), as well as spelling–sound consistency effects and natural variation in nonword pronunciation (Zevin & Seidenberg, 2006). Lesioning of semantic contributions in an ANN model also replicates the symptoms of surface dyslexia, as seen in participants with semantic dementia (Woollams et al., 2007). ANN models, however, have been shown to perform at a below-human level on low-frequency, inconsistent words (Jared, 2002). In contrast, the DRC model succeeds in accounting for the serial, left-to-right nature of reading English (Coltheart et al., 2001). The DRC model is silent, however, on effects of semantic factors such as imageability, the degree to which a word is judged to elicit a sensory impression (Plaut & Shallice, 1993; Strain & Herdman, 1999; Strain et al., 1995), although such effects are beyond the scope of the current investigation.

Both ANN models (Ueno et al., 2011) and the DRC model (Perry et al., 2007, 2010) have been expanded in their capacity to simulate reading behavior since they were originally introduced. Here we elected to use relatively simple versions of the models that are maximally comparable.

Some previous studies have used computational models of reading behavior to inform computational models of neural processing. A series of studies has used ANN models of reading to examine the N400 event-related potential component, thought to index attempted semantic access. Broadly, these results suggest that ANN models with neurobiologically inspired architecture can produce realistic N400 waveforms, account for waveform-modulating variables such as frequency or semantic richness, and perform lexical decision tasks (Cheyette & Plaut, 2017; Laszlo & Armstrong, 2014; Laszlo & Plaut, 2012; Rabovsky et al., 2018).

The present study differs from this earlier work in several ways. First, previous attempts to directly connect models of reading with neuroimaging data have been limited to electrophysiological data. We aimed to extend this literature to fMRI-based word representations. Second, previous work has compared model outputs with neural data, rather than model internal representations. Our approach focused on what the model is computing by examining the intermediate stage (hidden layer) associated with orthography-to-phonology transforms, as opposed to dealing solely with the output representations. Finally, and most importantly, previous studies have not directly compared representations from both the DRC and ANN models in terms of their fit to neural data. Instead, they have demonstrated that computational mechanisms incompatible with the DRC model can produce a qualitative fit to neural data. We aimed to determine not only whether it is possible to produce a qualitative fit to neural data, but also whether either model produces a better quantitative fit.

Interest in using computational models of reading to interpret neural data on reading is long-standing. Even early functional neuroimaging studies of reading interpreted their results in terms of the ANN and DRC models (Binder et al., 2005; Fiez et al., 1999). Furthermore, in the decades since these models were developed, a considerable amount has been learned about the neural basis of reading. Successful reading involves the integration of visual, orthographic, phonological, and semantic information. Both univariate and multivariate pattern analyses (MVPA) have contributed to localizing and dissociating where in the brain these types of information are processed (Binder et al., 2009; Fischer-Baum et al., 2017; Graves et al., 2010; Liuzzi et al., 2017; Price, 2012; Taylor et al., 2013).

Perhaps the most consistent finding in imaging studies of reading concerns the involvement of the left mid-fusiform gyrus (FG), also referred to as the occipitotemporal cortex (Price, 2012), in orthographic processing. The FG is thought to be an initial part of the pathway from orthography to phonology when reading (Graves et al., 2014; Jobard et al., 2003). Activity in the FG has been shown to follow a spatial gradient, with greater activation to lower-level visual and smaller orthographic features (e.g., single letters or bigrams) in the posterior aspect and tuning to progressively larger orthographic structure (e.g., quadrigrams or whole word forms) in more anterior aspects (Vinckier et al., 2007). The FG shows increased activation as a result of grapheme–phoneme correspondence (GPC) training in children (Brem et al., 2010; Shaywitz et al., 2004) and adults learning novel writing systems (Hashimoto & Sakai, 2004; Taylor et al., 2017). Furthermore, this increased activation to letter strings and words fails to develop in children with dyslexia (Shaywitz et al., 2007; van der Mark et al., 2009), and damage to the mid-FG results in pure alexia (Binder & Mohr, 1992; Damasio & Damasio, 1983; Leff et al., 2001). The mid-FG is sensitive to letter combination probabilities, even when the stimuli are nonpronounceable letter strings, and engages in orthographic processing even when participants are attending to nonlinguistic visual features of the stimuli (Binder et al., 2006). Despite this, the exact computational role of the FG in reading is unclear. The visual word form area hypothesis posits that the FG is specialized for orthographic processing (Cohen et al., 2000; Dehaene & Cohen, 2011; McCandliss et al., 2003). The Interactive Account, on the other hand, suggests that the role of the FG is to integrate phonological and possibly semantic information with bottom-up visual and combinatorial orthographic information (Mano et al., 2013; Price & Devlin, 2011; Twomey et al., 2011). A model-based approach with separate representations for orthography, phonology, and the mapping between them should help clarify the computational role of the FG.

Semantic information is theorized to assist in producing phonology during reading aloud, particularly when reading inconsistent or irregular words (Strain et al., 1995). We briefly summarize relevant information on this critical component of reading. However, strong conclusions regarding semantics are beyond the scope of this study due to the lack of specific semantic representations in the models being tested. Investigations of activation related to semantics in reading reveal a specific, yet widespread network, prominently including the angular gyrus (AG), along with middle and anterior parts of the lateral and ventral temporal cortices (Binder et al., 2009; Taylor et al., 2013). The AG has been frequently implicated in semantic processing (Binder et al., 2009; but see Humphreys et al., 2015). While its exact role is debated, the AG has been associated with semantic integration (Humphries et al., 2007), processing of semantic features (Binder & Desai, 2011; Wang et al., 2017), more general effects of task difficulty (Humphreys et al., 2015), and overlapping effects of semantics and task difficulty (Mattheiss et al., 2018). The lateral and ventral temporal cortex are involved in the storage and processing of category-related semantic information (Lambon Ralph et al., 2017). Broad swathes of temporal cortex show activation in categorization tasks (Chao et al., 1999; Kable et al., 2005), and lesions result in category-specific processing impairment (Damasio et al., 2004; Hillis & Caramazza, 1991; Lambon Ralph et al., 2007). The left anterior temporal lobe (ATL), though it has been suggested to be part of the default mode network (Buckner et al., 2008; Raichle et al., 2001), demonstrates increased activation compared with rest across stimulus type and modality in semantic tasks (Humphreys et al., 2015). The left ATL has been theorized to map multimodal semantic representations onto phonology (Hoffman et al., 2015).

Phonological information is processed in a cortical network largely distinct from those for orthography and semantics, involving the left posterior superior temporal gyrus (pSTG), the supramarginal gyrus (SMG), and the pars opercularis of the inferior frontal gyrus (IFG). This network is broadly activated when mapping orthography directly onto phonology (Graves et al., 2010; Jobard et al., 2003; Sandak et al., 2004). Supporting this, the pSTG, SMG, and IFG pars opercularis also show decreased activation during reading in subjects with dyslexia (Richlan et al., 2009). Lesions of the SMG have also been shown to produce conduction aphasia (Damasio & Damasio, 1980) and phonological agraphia (Alexander et al., 1992). Deficits in phonological retrieval, as distinct from semantics or comprehension, have also been associated with left-sided lesions focused on the pSTG and SMG (Pillay et al., 2014). Furthermore, an fMRI meta-analysis examining encoding and recall of phonological stimuli found an area of maximum overlap with lesions producing conduction aphasia in the planum temporale, just inferior of the SMG (Buchsbaum et al., 2011). For reading in particular, decreased bigram frequency, likely reflecting difficulty of mapping orthography to phonology, has been related to increased activation in bilateral superior temporal sulcus and posterior middle temporal gyrus (MTG) (Graves et al., 2010).

In addition to the largely localized perspectives on the neural basis of orthographic, semantic, and phonological processing given above, MVPA studies have begun to unravel the structure of neural representations related to these processes. Rothlein and Rapp (2014) demonstrated the presence of separate neural substrates for modality-specific and abstract representation of letters. Recent studies have also found category-specific tuning in the ATL (Malone et al., 2016), semantic representations in the FG (Fischer-Baum et al., 2017; Wang et al., 2018), and orthographic representations in the AG (Fischer-Baum et al., 2017). However, despite these great strides in mapping the neural basis of some of the major cognitive components of reading, there is little consensus on the neural computations these representations may reflect.

### The Current Study

The present study aimed to provide the first direct comparison of how well the ANN and DRC models of reading fit fMRI data. Historically, the difficulty of bringing model-based representations and neural representations into the same space prevented direct comparisons. Representational similarity analysis (RSA) provides an elegant solution to this problem (Kriegeskorte & Kievit, 2013; Kriegeskorte et al., 2008). RSA enables the comparison of representations from disparate modalities by transforming those representations into a common space based on stimulus similarity. Using RSA, we related neural data from human participants reading aloud to the internal representations generated by the ANN and DRC models performing the same orthography-to-phonology task. We predicted that both models of reading would correspond to neural activity in a left-lateralized reading network spanning frontal, temporal, and fusiform gyri. These cortical regions have been repeatedly implicated in studies of reading (Binder et al., 2009; Cattinelli et al., 2013; Fiez & Petersen, 1998; Murphy et al., 2019; Price, 2012; Taylor et al., 2013; Turkeltaub et al., 2002). Furthermore, due to the neurally inspired nature of its architecture and function, including its use of distributed as opposed to localist symbolic representations, we predicted that the ANN model distributed representations would provide a better fit for investigating how the brain computes orthography-to-phonology transforms. In comparing the neural instantiation of the ANN and DRC models, we not only tested their feasibility as mechanistic explanations of reading, but also demonstrated a principled methodology for comparing computational models of cognition.

## MATERIALS AND METHODS

### Participants

The participants were 18 (13 female) healthy, right-handed adults who spoke English as a first language. The mean age of the participants was 23.2 (*SD*: 3.4). Subjects provided written informed consent following procedures approved by the Medical College of Wisconsin Institutional Review Board, as described in Graves et al. (2010).

### Stimuli

464 monosyllabic English words were used as stimuli. These words were selected such that letter length, word frequency, spelling–sound consistency, imageability, bigram frequency, and biphone frequency were uncorrelated. Further details regarding the stimuli can be found in Graves et al. (2010). One word, “hale,” was discarded from the original 465 word stimulus list because it was not in the Harm and Seidenberg (2004) stimulus set. This was done to ensure compatibility with planned studies investigating the contribution of semantics to models of reading.

### Task

The fMRI task used a fast event-related design with continuous acquisition. Participants viewed a series of randomly presented words. Each word was displayed for 1,000 ms before being replaced by a fixation cross. Participants were instructed to “read each word aloud as quickly and accurately as possible” into an fMRI compatible microphone.

The MRI data were acquired using a 3T GE Excite system (GE Healthcare, Waukesha, WI) using an 8-channel array head radio frequency receive coil. A 134 contiguous axial slice (0.938 × 0.938 × 1.000 mm) T1-weighted anatomical image was acquired using a spoiled-gradient-echo sequence. Functional scans were acquired using a gradient-echo echoplanar imaging (EPI) sequence (echo time = 25 ms, repetition time = 2,000 ms, field of view = 192 mm, matrix = 64 × 64 pixels, voxel dimensions = 3 × 3 × 2.5 mm, gap = 0.5 mm) in an interleaved fashion, resulting in thirty-two axial slices per volume. 240 volumes were collected in each of five runs.

### Orthographic and Phonological Representations

The orthographic representations, used as inputs to the ANN model, were the inputs used in Plaut et al. (1996, Table 2). They consisted of 105-unit binary vectors. The presence or absence of a grapheme was indicated with a 1 or 0, and the graphemes were grouped according to whether they could appear in the onset (first 30 units), vowel (next 27 units), and coda (final 48 units). Multi-letter graphemes such as “ph” were coded such that “p,” “h,” and “ph” were all set to 1. An orthographic representational dissimilarity matrix (RDM) was computed as the pairwise correlation distance between all orthographic vectors for later use in RSA (see supplementary Figure 1 in the online supporting information located at https://www.mitpressjournals.org/doi/suppl/10.1162/nol_a_00018). The phonological representations, used as the target outputs for training and testing the ANN models, were also binary vectors as used in Plaut et al. (1996, Table 2). They consisted of 61 phoneme slots, also divided into onset, vowel, and coda, where 1 indicated the presence and 0 the absence of a phoneme. The phonemes /ps/, /ks/, and /ts/ were also coded such that both their constituent parts and the combination unit were active. A phonological dissimilarity matrix was computed as the pairwise correlation distance between all phonological vectors. (See supplementary Figure 1 in the online supporting information.)

### Computational Models

**Figure 1.**
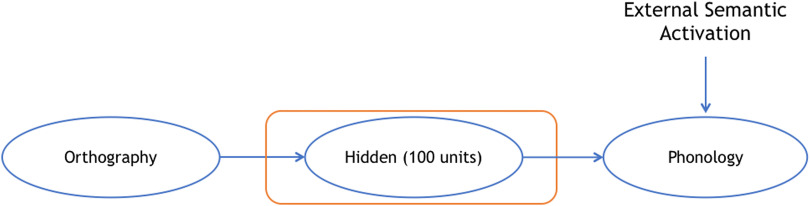
Schematic structure of the artificial neural network model. Word representations for the model were derived from the hidden layer, circled in orange.

Two computational models, the feed-forward ANN from Plaut et al. (1996) and the rule-based, symbolic DRC model (Coltheart et al., 2001), were used to simulate human reading. The ANN model was identical to the one in Plaut et al. (1996). The models were trained with inputs, described above, representing 2,998 monosyllabic words. The model had 105 input units. These input units were fully connected to 100 hidden units, which in turn were fully connected to 61 phonological output units (Figure 1). These phonological units were given additional, external input that ramped up as training proceeded. This external input was calculated as a function of the log-compressed frequency for a given word (Plaut et al., 1996, Equation 16), and approximates the growing semantic contribution to phonology that occurs over the development of reading. It further reflects the assumption that higher frequency concepts have stronger semantic representations (Plaut et al., 1996; Woollams et al., 2007). Finally, using frequency as a simplified approximation for semantics also ensures that the ANN model is maximally comparable to the DRC model, which also does not try to account for detailed semantics.

The model was trained for 400 epochs (i.e., 400 presentations of the entire 2,998-word stimulus set) using standard backpropagation of error and tested for generalization on a separate set of nonwords. The number of epochs was chosen using Plaut et al. (1996) as a starting point and then fine-tuned to identify the point at which accuracy on a nonword test set began to decrease. The test set contained 166 monosyllabic nonwords, compiled from Glushko (1979) and McCann and Besner (1987). Accuracy on the training set was assessed by testing the model using the 464 words that the human participants read in the scanner. These words were a subset of the full 2,998-word training corpus. Outputs from the network were rounded to the nearest whole number (either 0 or 1) and compared with the appropriate target vector. As real words have well-defined pronunciations, any network output vector that deviated from the appropriate target vector was marked incorrect.

Nonwords, however, do not have a single correct pronunciation. To account for this, a list of plausible alternative pronunciations was generated. For scoring the test set, outputs from the network were rounded to the nearest whole number (either 0 or 1) and compared with the full list of target vectors using the Jaccard similarity index (Levandowsky & Winter, 1971). If the output vector produced by the network was closer to the appropriate target vector than to any other vector in the nonword set, the output was marked correct. If the output vector was closer to the vector of any nonword other than the target, the output was marked incorrect. This reflects the intuition that nonwords do not have well-defined pronunciations. However, it allows for the model to have a reasonably random output, so long as that output is close enough to one of the acceptable pronunciations. Thus, we also tested a stricter scoring method. Rounded model outputs for a given nonword were compared with the list of acceptable pronunciations for that nonword. If the output exactly matched one of these vectors, it was marked correct. The model was run 20 times, with weights re-initialized each time to random uniform values between −0.1 and +0.1. We chose 20 instantiations of the model to roughly match the number of participants in the fMRI data set. This number was expected to be ample, as it is twice that used in similar previous modelling experiments (Welbourne & Lambon Ralph, 2005; Woollams et al., 2007). The stimulus-stimulus correlation matrices generated from the hidden unit layers of these model instantiations were averaged to create a mean ANN RDM.

**Figure 2.**
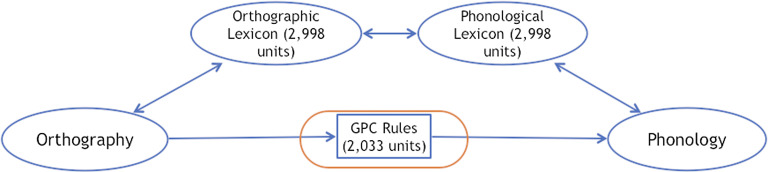
Schematic structure of the dual-route cascaded model. Word representations for the model were derived from the graphene-phoneme correspondence (GPC) rules, circled in orange.

The second model was the symbolic, rule-based DRC model (Coltheart et al., 2001). Inputs to the DRC model activated visual feature units, which then fed forward to activate letter units. The activation from this letter unit layer then activated two routes in parallel: a lexical-nonsemantic route and a GPC route (Figure 2). The lexical-nonsemantic route contained an orthographic lexicon, in which a local word representation was activated by the parallel activation of earlier letter features. This orthographic lexicon activation then activated the corresponding phonological lexicon representation of the input word. Each of these lexicons had 2,998 units, one for each word in the current corpus. The output of the lexical-nonsemantic route consisted of the model activation value in the unit corresponding to the input word and a 0 in every other position, for each lexicon. The GPC route processed each input word in a serial, left-to-right fashion. When the first letter of an input is processed, the 2,033 preset rules are searched until an appropriate letter-phoneme conversion is located. The next letter then becomes available to the GPC route, and the two-letter string undergoes the same search through rule space. At each stage, rules are searched from largest to smallest matching grapheme. This process is repeated until the input word is named or until the last letter is processed. The output of the GPC route consisted of the model activation for each rule activated by the input word, with a 0 in every other position. Word representations for the DRC model were taken as the vector of activation for the GPC rules. Deriving word representations from these internal components of the model was considered analogous, for purposes of comparison, to representations derived from the hidden unit layer of the ANN. This internal DRC representation consisted of a 2,033-unit sparse vector. Further details of the DRC model can be found in Coltheart et al. (2001).

An important feature of the DRC model is that most words are read by both the lexical and GPC routes, with the GPC route being primary only for unknown words, nonwords, or low frequency regular words (Coltheart et al., 2001). The lexical route has no direct analogue in the ANN model. However, it is possible that representational similarities between the brain and the model might be driven by the lexical route. As such, we also considered 8,029 unit DRC representations (2,033 GPC + 2,998 orthographic lexicon + 2,998 phonological lexicon units), wherein the lexicon representations consisted of a one-hot, localist vector for each word. As the results with this expanded representation (see Supplementary Figure 3 and Supplementary Table 1 in the online supporting information) were nearly identical to the results that did not include the lexical route, we chose the parsimonious approach of focusing on the GPC route, as it is most analogous to the processes in the ANN model. Therefore, we do not consider the lexical route representations further.

### fMRI Data Analysis

MRI data were preprocessed using AFNI (http://afni.nimh.nih.gov/afni; Cox, 1996). Data were skull-stripped, slice-timing and motion corrected, and the first six images were discarded to allow for initial saturation. Data were then spatially coregistered.

Voxelwise single-trial effects were estimated using least-squares-sum multiple regression (Mumford et al., 2012), as implemented in the AFNI program 3dLSS. A noise signal calculated from the signal in the lateral ventricles was included along with six motion parameters as covariates of no interest. Resulting coefficient maps were not smoothed during first-level analysis, so as to preserve spatial patterns for RSA analysis. Individual subject data were then transformed into Talairach space (Lancaster et al., 2000).

### RSA (Sensitivity Analysis)

To test for correspondence between model-based and brain-based similarity patterns among the stimuli, RSA was performed. This was implemented using PyMVPA software (Hanke et al., 2009). For each model, stimulus features were *z*-scored. An RDM was then generated based on the pairwise correlation distance (1 − Pearson’s *r*) between all stimuli. To test for correspondence between this RDM and neural patterns, a searchlight analysis was performed. Each voxel in a cortical gray matter mask iteratively served as the center of a 3-voxel radius sphere (sphere volume: 123 voxels of 3 × 3 × 3 mm each, including the center voxel). A neural RDM was constructed by calculating the pairwise correlation between the beta weights evoked from each stimulus in this sphere. This neural RDM was then compared with the model RDM using Spearman correlation. Use of a rank-based second-order correlation avoids potential differences in correlation means across different types of representations, as recommended by Kriegeskorte et al. (2008). The resulting correlation coefficient was assigned to the center voxel of each sphere. This process was repeated until every voxel in the gray matter mask served as a center voxel once. The individual subject’s resulting correlation coefficient maps were spatially smoothed using a 6 mm full-width-half-max (FWHM) kernel before being entered into a one-sample *t* test against a null hypothesis of no correlation without further resampling. The resulting group maps were Fischer transformed and submitted to cluster correction (voxelwise *p* < 0.001, clusterwise *p* = 0.05, cluster extent = 234.9 mm^3^).

### Partial Correlations

Separate partial correlations were calculated using the CoSMoMVPA package for MATLAB (Oosterhof et al., 2016). The correlation coefficient maps were then smoothed with a 6 mm FWHM kernel, and *z*-scored. A *t* test was run on the *z*-score maps, and the group *t*-value map was subjected to cluster correction (voxelwise *p* < 0.001, clusterwise *p* = 0.05, cluster extent = 234.9 mm^3^).

## RESULTS

### Human Performance

The mean reaction time was 588 ms (*SD*: 123). Errors in reading aloud were very rare, only 2.6% overall. Further details can be found in Graves et al. (2010).

### Model Performance

To determine whether the 20 instantiations of the ANN model achieved an accuracy comparable to human performance, they were tested, as described in Materials and Methods, for the 464 words that humans read in the scanner. Those were a subset of the 2,998 words on which the model was trained. The model obtained 97.2% accuracy on this subset. To test the ability of the models to generalize beyond the training set, they were presented with novel but pronounceable letter strings (hereafter, nonwords). Using Jaccard similarity as a scoring metric, the ANN model attained 76.9% accuracy on the Glushko (1979) nonwords and 91.9% accuracy on the McCann and Besner (1987) nonwords, reaching 84.9% mean accuracy for nonwords overall. Using the stricter exact-match criterion, the ANN was 75.5% accurate on the Glushko (1979) nonwords and 79.5% accurate on the McCann and Besner (1987) nonwords, achieving 77.4% mean overall accuracy. The DRC model made no errors on either nonword set. The dissimilarity matrices derived from the intermediate representations of the ANN and DRC models were significantly correlated (*r* = 0.52, *p* < 0.0001; Table 1). (See Supplementary Figure 2 in the online supporting information for plots of example and full ANN and DRC dissimilarity matrices.)

**Table 1.** Correlation matrix of the dissimilarity matrices used for representational similarity analysis.

	ANN	DRC	Orthography	Phonology
ANN	1			
DRC	0.52	1		
Orthography	0.9	0.55	1	
Phonology	0.68	0.6	0.63	1

*Note*. ANN = artificial neural network, DRC = dual-route cascaded model.

### Imaging Results

Here we focus on comparing model-based word representations with neural representations derived from functional neuroimaging. Univariate analyses of neural activation for these words are reported in Graves et al. (2010) and will not be discussed further.

#### Orthography

**Figure 3.**
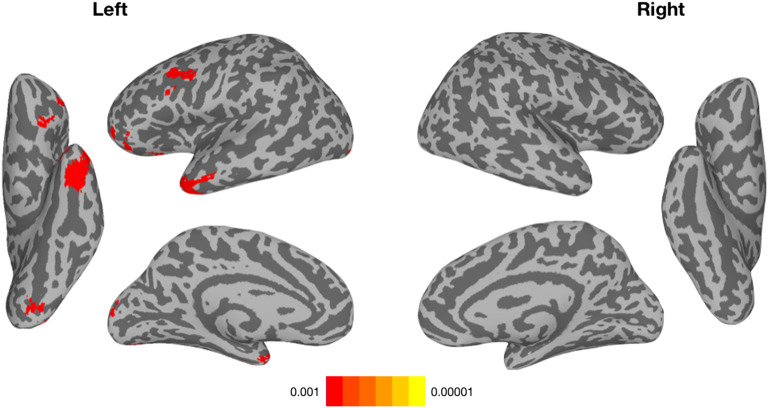
Correspondence between the orthographic input similarity structure and the neural similarity structure.

To test for neural correspondence with the orthographic representations used as inputs to the ANN model, a searchlight RSA was performed. The orthographic input RDM was correlated with neural representations in a left-lateralized network. Specifically, orthographic features correlated with neural activity in the left ATL (lateral and ventral aspects), the orbital IFG, the middle frontal gyrus, the precentral sulcus, the collateral sulcus, the calcarine fissure, and the cuneus. The orthographic RDM was also correlated with neural activity to a lesser extent in the cerebellar tonsil (Figure 3 and Supplementary Table 2 in the online supporting information).

#### Phonology

**Figure 4.**
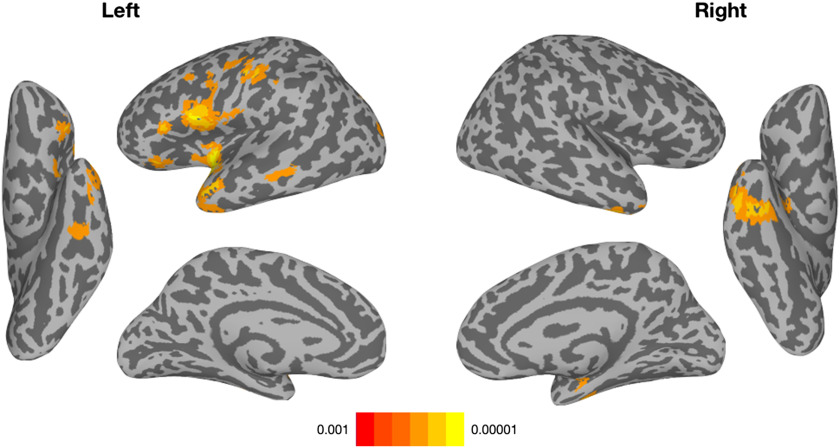
Correspondence between the phonological output similarity structure and the neural similarity structure.

Neural correspondence with the phonological ANN output feature representations was also tested using searchlight RSA. Phonological feature representations were correlated with neural representations in the left middle temporal and anterior superior temporal gyri, the middle occipital gyrus, the insula, and the pre- and postcentral gyri. Phonological representations were also related to bilateral neural activity in the anterior inferior temporal and parahippocampal gyri (Figure 4 and Supplementary Table 3 in the online supporting information). Importantly, the phonological searchlight did not reveal any significant clusters in the primary visual cortex. This points to the external validity of the current approach, where correspondence between model and neural representations is able to clearly distinguish between orthographic inputs and phonological outputs in expected brain areas.

#### Intermediate Representations from Models

**Figure 5.**
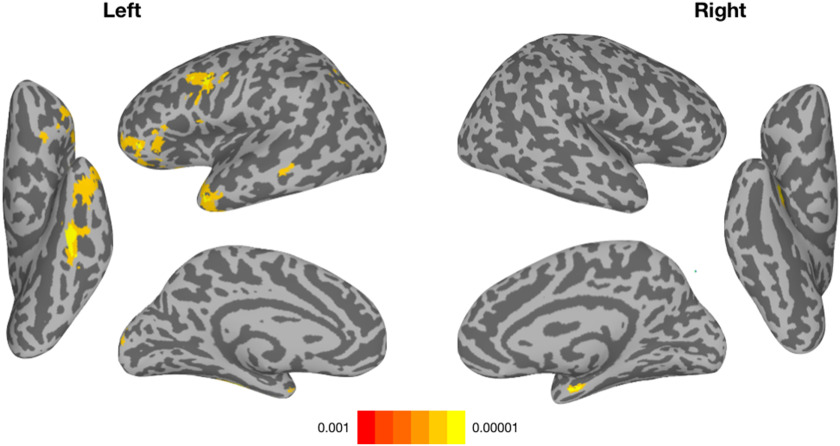
Correspondence between the artificial neural network hidden layer similarity structure and the neural similarity structure.

The hidden layer of the ANN was used to model information related to the transformation of orthography to phonology. The RDM constructed from model activations in this hidden layer correlated with left hemisphere neural representations in the anterior temporal pole, the orbital and triangular IFG, the precentral sulcus, the FG, the intraparietal sulcus, the cuneus, and the MTG (Figure 5 and Supplementary Table 4 in the online supporting information). Additionally, the hidden layer RDM correlated with right hemisphere representations in the parahippocampal gyrus. This pattern of results largely agrees with a standard neuropsychological view, in which reading aloud is supported by a combination of visual-related areas in the occipito-temporal cortex, language production areas in the left IFG, and intermediate areas along the left MTG (Binder et al., 2009; Fiez & Petersen, 1998; Price, 2012; Taylor et al., 2013; Turkeltaub et al., 2002). At the same time, our approach lends a new level of specificity to the functions being carried out in these areas during reading.

**Figure 6.**
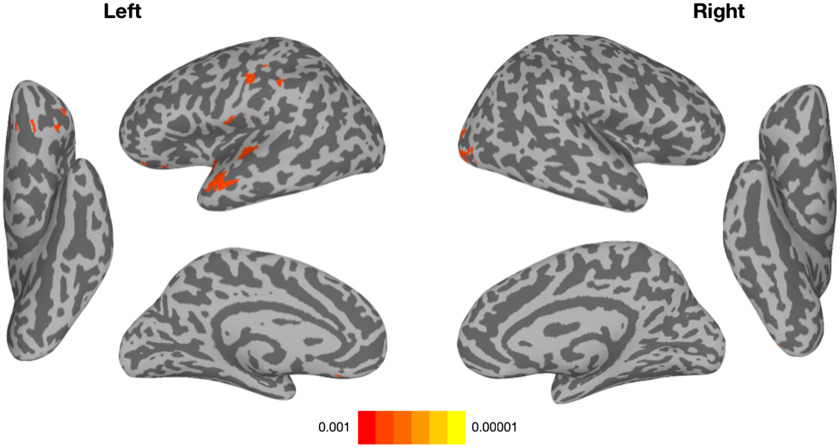
Correspondence between the dual-route cascaded intermediate graphene-phoneme correspondence route similarity structure and the neural similarity structure.

As our aim was to model the mapping between orthography and phonology, we elected to use representations generated from the DRC model layer that specifically implemented this process: the GPC rule route (Figure 2). This route also provides the best match to the function of the hidden layer of the ANN model (Figure 1). The RDM based on GPC representations from the DRC model was correlated with a more restricted set of largely left hemisphere representations in the superior temporal gyrus and sulcus, the postcentral gyrus, the straight gyrus, and the IFG pars orbitalis (Figure 6 and Supplementary Table 5 in the online supporting information). In the right hemisphere, the DRC RDM correlated with the neural representation in the inferior occipital gyrus. These neural correspondences with the intermediate GPC level of the DRC are spatially restricted relative to those found for the ANN, and they appear in areas less typically related to language and reading. This suggests that the ANN model might provide a better fit to the neural data than the DRC model.

#### Partial Correlations between Models

**Figure 7.**
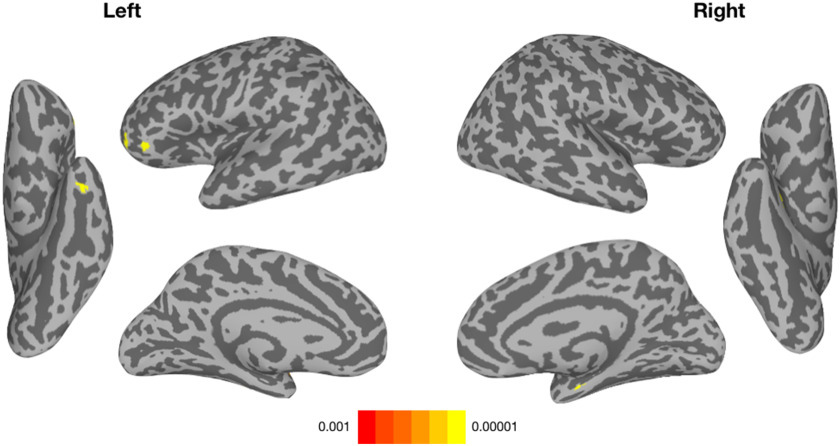
Correspondence between the artificial neural network hidden layer similarity structure and the neural similarity structure with variance due to the dual-route cascaded model partialled out.

To formally test the ability of each model to account for the neural data after accounting for the other, we used partial correlations. This approach also accommodates the moderate correlation between the ANN and DRC intermediate layer representations. A searchlight analysis correlating the intermediate layers of the DRC model to the neural data with the variance due to the ANN hidden layer RDM partialled out revealed no results. However, when the reversed analysis was conducted—the ANN hidden layer RDM correlated to the neural data with the variance due to the DRC model partialled out—a primarily left-lateralized set of areas emerged. These include the middle frontal gyrus, the orbital IFG, and the anterior inferior temporal gyrus. The ANN representations with variance due to the DRC model removed also correlated to neural activity in the right parahippocampal gyrus (Figure 7 and Supplementary Table 6 in the online supporting information). These results are consistent with the hypothesis that the distributed, nonsymbolic nature of the representations generated by the ANN model of reading reflects neural information processing to a greater degree than the symbolic representations of the DRC model.

## DISCUSSION

While there is broad agreement as to where in the brain written language is processed, what is being coded or represented in these regions has remained largely unclear. Existing computational cognitive models that account for reading behavior use theories containing very different kinds of representations. ANN models use distributed nonsymbolic representations, while the DRC model uses local symbolic representations. We used RSA to relate neural data obtained during reading to the representations of orthography-to-phonology transforms generated by these two distinct computational models. The results suggest that both the DRC and the ANN models correspond to neural activity related to transformations from orthographic to phonological representations. Consistent with our predictions, we also show that the distributed ANN model of reading reflects the neural activity involved in reading aloud to a greater degree than the symbolic DRC model.

Importantly, the orthographic and phonological representations used as inputs to and outputs from the computational models correlate with neural activity in relevant cortex. The orthographic representations correspond to a network of cortical regions commonly associated with reading (Price, 2012; Turkeltaub et al., 2002). This network spans frontal, temporal, and occipital cortex. Unexpectedly, orthographic inputs did not correlate with neural representations in the posterior occipito-temporal sulcus typically associated with orthographic processing. We used grapheme-level orthographic representations, consistent with previous ANN models (Plaut et al., 1996; Woollams et al., 2007). However, graphemes contain clusters of letters such as “th,” meant to more closely correspond to phonology than would purely orthographic inputs. Hence, graphemic similarity structure across words may reflect higher-order associations beyond orthography.

Phonological representations also correlate with brain structures that have been shown to be sensitive to phonological structure, including the left hemisphere middle and superior temporal gyri, and the ATL (Hoffman et al., 2015; Richlan et al., 2009). The phonological similarity structure was also related to neural activity in the pre- and postcentral gyri, most likely reflecting word production (Guenther et al., 2006; Indefrey & Levelt, 2004).

Both models account for how orthographic inputs are transformed to phonological outputs. This transform is reflected in the hidden layer of the ANN model and the GPC route of the DRC model. These intermediate representations corresponded to neural representation in areas of the brain involved in reading. In particular, the neural correspondences with the hidden layer of the ANN model overlap heavily with those for the orthographic representations, showing clusters in IFG, MTG, and ATL. Many of these regions have been previously implicated in orthography-to-phonology transformation (Jobard et al., 2003). The ANN representations also corresponded with neural representation in the FG. While the exact role of the FG has been debated (Dehaene & Cohen, 2011; Price & Devlin, 2011), it has been consistently implicated in the reading of words. A recent study suggests that the middle (mFG) and anterior fusiform gyrus (aFG), but not the posterior FG, contain neural representations of both orthographic and phonological information (Zhao et al., 2017). Consistent with this finding, we also found partially overlapping representation of orthographic and phonological features in both the mFG and aFG. We also found correspondence between the ANN and the neural similarity structures in the mFG and aFG, further supporting the validity of the ANN hidden layer representations as an intermediate step between orthography and phonology. Abstractness of orthographic information, as indexed by invariance to case, font, and letter position (Dehaene et al., 2004), and representation of larger orthographic as opposed to low-level visual features, have been shown to increase along a posterior-to-anterior axis (Vinckier et al., 2007). Whereas the orthographic RSA identified clusters of correlation along the length of the FG, the hidden layer RSA reveals clusters in only the mFG and aFG, suggesting that the distributed representations generated from the model may indeed reflect larger and more abstract orthographic units. The ANN representations were also correlated with neural representations in the motor cortex, potentially reflecting a final transformation from the orthographic input to the motor code associated with a phonological output (Guenther et al., 2006).

The DRC model intermediate representations showed neural correspondences that spatially overlapped with the phonological representations used as target outputs in the ANN model in anterior temporal and Rolandic cortices. The DRC model intermediate representations also showed some overlap with the standard reading network (Binder et al., 2009; Cattinelli et al., 2013; Fiez & Petersen, 1998; Murphy et al., 2019; Price, 2012; Taylor et al., 2013; Turkeltaub et al., 2002) in the inferior frontal, the superior temporal, and the anterior temporal cortex. The ATL and the IFG pars orbitalis have been implicated in semantic processing (Binder et al., 2009; Hoffman et al., 2015) and the superior temporal gyrus in phonological processing (Fiez & Petersen, 1998; Price, 2012; Turkeltaub et al., 2002). This correspondence with reading-related areas was, however, more spatially limited than was found for the ANN model. Counter to our expectations, the DRC model did not correlate with activity in the FG, a putative locus of orthographic processing. The DRC model’s lack of correlation with cortex generally associated with orthographic processing may be due to the different, more localist form of the input, or it may point to the distributed nature of the neural representations reflected in the intermediate representations of the ANN but not the DRC model.

Despite the significant correlation between the representations generated by the models, partial correlation analysis revealed that the distributed representations generated by the ANN model reflect neural representations to a greater degree. The network of neural activity resulting from this analysis is spatially limited, but partially corresponds to the standard brain reading network. In particular, both the IFG pars orbitalis and the inferior ATL are implicated in semantic processing during reading (Binder et al., 2009; Hoffman et al., 2015). Additionally, lesions of these same cortical regions produce regularization errors, in which a regular spelling-to-sound correspondence is improperly applied to an irregular word, as in pronouncing “sew” to rhyme with “do” (Binder et al., 2016). Overall, partial correlation results suggest that the ANN model better captures the neural representations involved in transforming orthography to phonology.

A possible explanation for the ANN-to-neural correspondence being greater than the DRC-to-neural correspondence is the sparser, binary nature of the DRC representations. ANN representations are dense and take rational number values, and thus are natively more like fMRI data. However, in order to assess ANNs as a model of neural representation, it must first be established that (1) ANNs actually do learn structured representations analogous to those in the brain and (2) the fit of a representation derived from an ANN model does in fact correspond to neural data above and beyond a representation derived from a symbolic model. Our results suggest that the ANN model does learn representations similar to neural representations, and that those representations do fit neural data better than representations derived from the symbolic DRC model. In short, brain activity does not tend to resemble local, symbolic rules, but instead computations over distributed elements.

The similarity structure of neural activity in the left ATL was related to every representational similarity structure we tested. The exact role of the left ATL in reading is unclear, although it has been linked to both semantic and phonological processing. There is fMRI evidence in healthy participants that it maps between semantic and phonological representations, particularly when reading exception words (Hoffman et al., 2015). Evidence from lesion deficit studies, however, suggests a more direct role in producing phonology. Rudrauf et al. (2008) found that lesions to the left ATL produced deficits in naming, but not recognition, of pictures across a range of categories. The co-presence of sensitivity to orthographic and phonological information in the left ATL was unexpected. This result may suggest that the role of the ATL involves integration across multiple levels and types of reading-related representation. Further investigation is warranted to identify the exact role of the ATL in reading.

An interesting possibility arises from the DRC model’s conceptualization of spelling–sound knowledge as GPC rules. The similarity structures generated by the DRC will be sensitive to word regularity, or how well they correspond to the GPC rules (Coltheart et al., 2001). The ANN model instead exploits consistency, the statistical regularities between letter combinations and phonology, relative to the frequency of that spelling–to–sound correspondence in the entire corpus of words on which it is trained (Plaut et al., 1996). Evidence from reaction times suggest that consistency has a larger effect on reading performance (Cortese & Simpson, 2000; Jared et al., 1990), but independent effects of regularity have also been demonstrated (Glushko, 1979). It is possible that the correlation between the DRC model’s representations and neural activity reflects cortical sensitivity to regularity, whereas the correlation between the ANN model and neural activity reflects sensitivity to consistency. Future research should seek to disambiguate the neural correlates of consistency and regularity.

The internal representations generated by the two models were moderately and significantly correlated (*r* = 0.52, *p* < 0.0001). The strength of correlation was surprising, as the theoretical bases for the models are highly divergent. ANN models learn representations over the repeated presentation of stimuli, and they assume that many units are able to contribute to the representation of a given word and, furthermore, that these units reflect generalized lexical consistency. No single unit codes for a particular spelling-to-sound correspondence. Instead, the pronunciation common to a letter string shared across words is coded by multiple units (Seidenberg & McClelland, 1989). The DRC model, in contrast, assumes that once the model finishes cycling, only the units corresponding to the relevant symbolic rules and the corresponding lexical entry for a given input word will be activated (Coltheart et al., 2001). Thus, the correlation between the intermediate representations in these models may reflect that both types are successful at accounting for reading aloud, in that they ultimately represent valid phonological outputs for the input words. By analogy to Marr’s framework for levels of analysis in cognitive systems (Marr, 1982), these models differ at the level of their representations and algorithms, but their correlation may arise from solving the same specific computational problem of reading aloud. For example, the lexicons and grapheme–phoneme rules in the DRC model may represent a high-level interpretation of the more basic, neurally inspired mechanism instantiated in the ANN model. The presumably more basic mechanisms of the ANN model, on the other hand, may reflect areas of feature convergence, where intermediate blends of orthographic features are remapped to accommodate the statistical regularities of phonological output.

One potential reason that the ANN and DRC models showed fairly similar correspondence to neural representations is the steps taken to equate the model representations. The DRC model representations were derived from the GPC route, with the lexical route allowed to contribute to the final outputs but not considered in our main analysis (but see Supplementary Figure 3 and Supplementary Table 1 in the online supporting information for near identical results including the lexical route in DRC representations). To ensure fair treatment of both models, we limited the representations derived from the ANN model to a single hidden layer representing the orthography-to-phonology pathway, allowing external frequency-based sematic input to contribute to the model’s output but not directly affecting the hidden layer. There are clear extensions that can be made to the ANN model that are likely to improve correspondence to neural representations. Using deeper networks (networks with more hidden layers), potentially with neurobiologically inspired patterns of intra- versus interlayer connectivity (see Laszlo & Armstrong, 2014; Laszlo & Plaut, 2012), and adding recurrence are natural ways to improve the ANN model’s fidelity with neural processing. It is less clear how one would extend the DRC model to emulate the complexity of the brain. Furthermore, a plethora of work in the connectionist tradition has examined potential semantic route implementations for ANN models of reading (Harm & Seidenberg, 2004; Monaghan et al., 2017; Ueno et al., 2011); we are unaware of any work that develops the dual-route model’s semantic pathway. In this sense, enforcing fair comparison between the models has actually limited the fair treatment of the ANN model with respect to its correspondence with neural representation.

These results support ANNs with distributed representations as a preferred model for the neural processing of reading. While the symbolic representations from the DRC model do correlate with neural activity, the effect is spatially restricted compared with the RSA map from the ANN representations. More stringently, removing the variance due the ANN model reveals that the DRC model corresponds to neural representations in no additional brain areas. This suggests that word representations in the brain are coded as distributed patterns of activation and are more sensitive to spelling–sound consistency than to rule-based regularity. The fact that the DRC model explains no neural variance above and beyond that of the ANN model also may suggest that the lower-level features of the ANN account for the GPC rules of the DRC model to some degree.

More generally, our results demonstrate the promise of directly comparing and adjudicating between computational models using neural data. While behavioral evidence alone has been insufficient for arbitration, RSA allowed us to compare the neural plausibility of predictions made by the ANN and DRC models. The correlation of the ANN model with a large number of relevant brain areas, and its ability to explain neural variance beyond that of the DRC model, demonstrate that the ANN model holds particular promise for connecting cognitive and neural approaches to reading in a computationally principled way. This method is simple to extend to any computational model that produces explicit, quantitative predictions.

One limitation to this study is the approximation of semantics used in the ANN model. While using frequency as a proxy for semantics reflects the intuitive idea that more common words are more likely to be understood, frequency is not a true semantic variable. Most notably, it ignores any effects that might vary based on the actual semantic content of words. For example, imageability has been shown to affect word reading speed and accuracy (Strain et al., 1995) and has distinct neural correlates compared to consistency and frequency (Graves et al., 2010). However, this frequency manipulation has been successfully used as an approximation for semantic input in multiple previous studies using the same model architecture used in this study (Plaut et al., 1996; Woollams et al., 2007). We plan to expand the ANN model to include a fuller implementation of semantics in future studies.

Exactly what the nature of these semantic representations should be remains to be determined. Optimization of hardware and advances in machine learning techniques have permitted modelling language at a larger scale than ever before. The recent success of transformer models suggests that computations over word co-occurrence and position are sufficient to generate relatively comprehensible and syntactically correct sentences (Vaswani et al., 2017). Indeed, co-occurrence based models are capable of predicting human behavior across a wide range of semantic tasks (Hofmann et al., 2018; Mandera et al., 2017; Pereira et al., 2016). However, a growing body of evidence from the embodied or grounded cognition literature suggests that neural representations of body states, emotions, and somatosensory experience play a role in how the brain computes word meaning (Barsalou, 2008). Both the co-occurrence and embodied feature approaches appear to be relevant to how the brain represents semantics. This was shown in a study using a word familiarity task in fMRI, which demonstrated distinct neural correlates of co-occurrence based and abstract semantic feature based (including embodied feature) representations of abstract words (Wang et al., 2017). Furthermore, context is known to have an effect on the processing of semantics, such as when disambiguating the meaning of homonyms (Rodd, 2020). As such, an online semantic subnetwork accounting for embodied features, co-occurrence, and context is a likely candidate for producing the most cognitively relevant semantic representations.

A second limitation is the nature of the phonological representations. Phonology is coded here as binary units, representing the presence or absence of a phoneme. Spatially, the phonological vector representation is subdivided into three sections corresponding to the onset, vowel, or coda of a word. This coding scheme has been used productively in previous modelling work (Plaut et al., 1996; Woollams et al., 2007), but is by no means a perfect all-purpose representation. It captures phoneme-level similarity (e.g., “cat” and “hat” are more similar than “cat” and “dog”) but cannot capture the more fine-grained differences that phonetic features might (e.g., “/p/” and “/b/” are exactly as similar as “/p/” and “/a/”). While our results agree with neurobiological models of phonology output, showing correspondence between the phonological representations and neural activity in the pre- and postcentral gyri (Indefrey & Levelt, 2004), future research could examine different phonetic or articulatory feature representations to determine which best fits the observed neural activity. In particular, it seems likely that more detailed phonological representations would improve correspondence with the middle and superior temporal gyri, which are sensitive to familiar phoneme patterns (Indefrey & Levelt, 2004; Price, 2012).

A final limitation is the performance of the ANN model on pronounceable nonwords. While the model did quite well, achieving a human-level accuracy of 97.2% on the tested real words, its 84.9% accuracy (77.4% by exact-match criterion) on the nonword test list remains somewhat below human-level performance. This issue may be partially addressed by extending the ANN model to more than one hidden layer. A single hidden layer allows for blends and partial combinations of features to be mapped to outputs, but multiple layers allow for combinations of those feature blends to be processed (LeCun et al., 2015). Deeper neural networks may not only improve performance on the nonword test set but may also better model the neural processes involved in reading.

Here we have directly compared two major computational cognitive models of reading in terms of their correspondence with neural data acquired during reading. The distributed ANN model accounted for unique variance beyond that of the DRC, revealing correspondence with neural representations in areas previously shown to be related to orthography or orthography–phonology mapping. Critically, correspondence of model representations with neural representations allows for the direct interpretation of neural patterns in terms of the computational function being implemented.

## ACKNOWLEDGMENTS

We thank Dr. Blair Armstrong for providing a version of the LENS neural network simulator that could be complied with later versions of Linux.

## FUNDING INFORMATION

William W. Graves, Eunice Kennedy Shriver National Institute of Child Health and Human Development (http://dx.doi.org/10.13039/100009633), Award ID: HD065839.

## AUTHOR CONTRIBUTIONS

William W. Graves developed the study concept and collected the data. Ryan Staples analyzed the data and drafted the manuscript. Both authors contributed to study design and the interpretation of the results. Both authors approved the final manuscript for submission.
