# Supplementary material for: Neural Components of Reading Revealed by Distributed and Symbolic Computational Models
Source: Neurobiol Lang (Camb). 2020 Oct 1;1(4):381–401. doi: 10.1162/nol_a_00018 (PMC9635488; doi:10.1162/nol_a_00018)
Supplement: Supplementary file 1 [file nol-1-4-381-s001.docx]

**Supplementary Material**


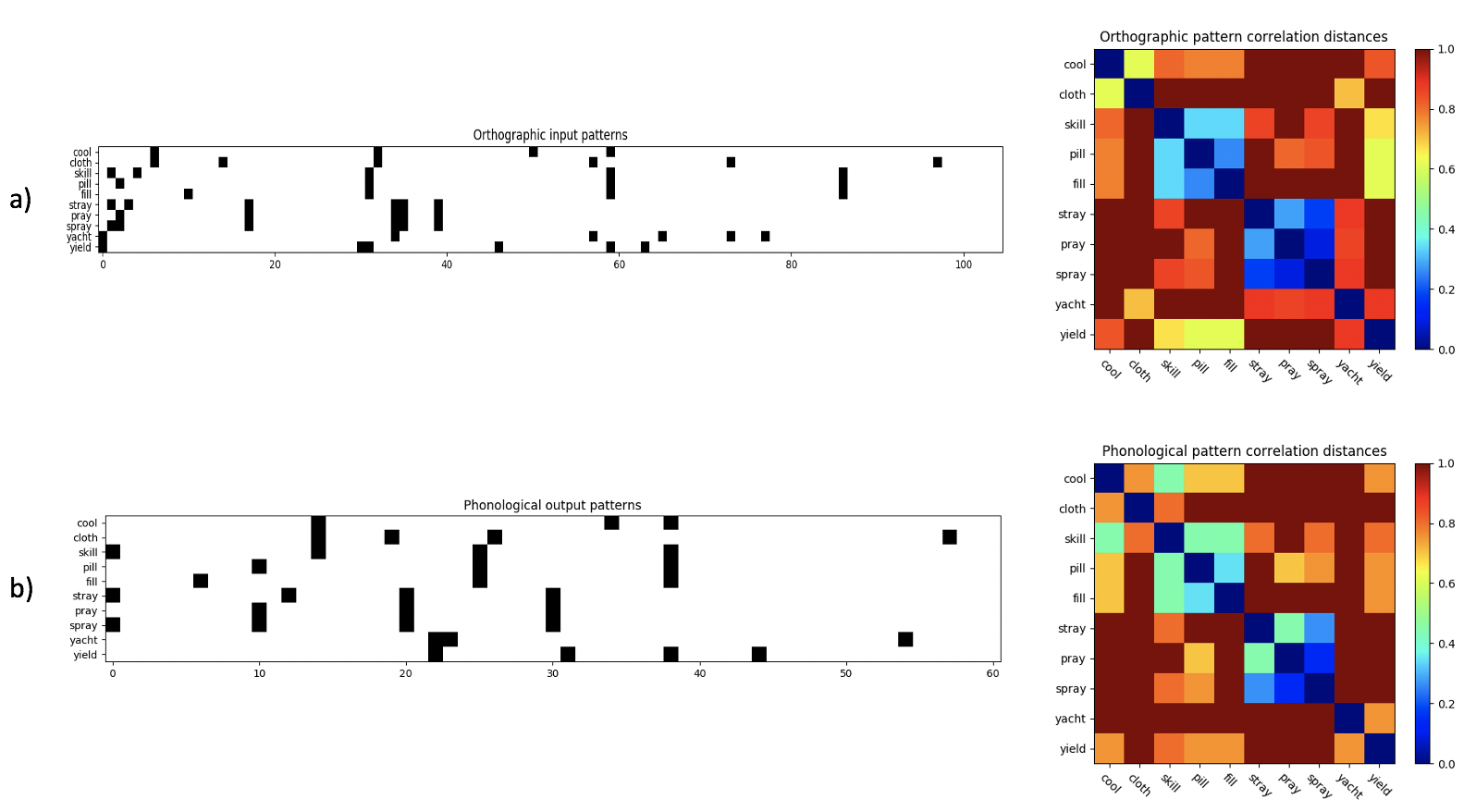


Supplementary Figure 1: a) Example orthographic vectors and orthographic dissimilarity matrix (DSM). b) Example phonological vectors and phonological DSM. Words chosen from the 464 word stimulus set that participants read in scanner. Two neighborhoods (skill/pill/fill, stray/pray/spray) were selected to demonstrate representation similarity within words that have similar orthography and phonology. These can be compared with cool/cloth and yacht/yield, which vary more along these dimensions.


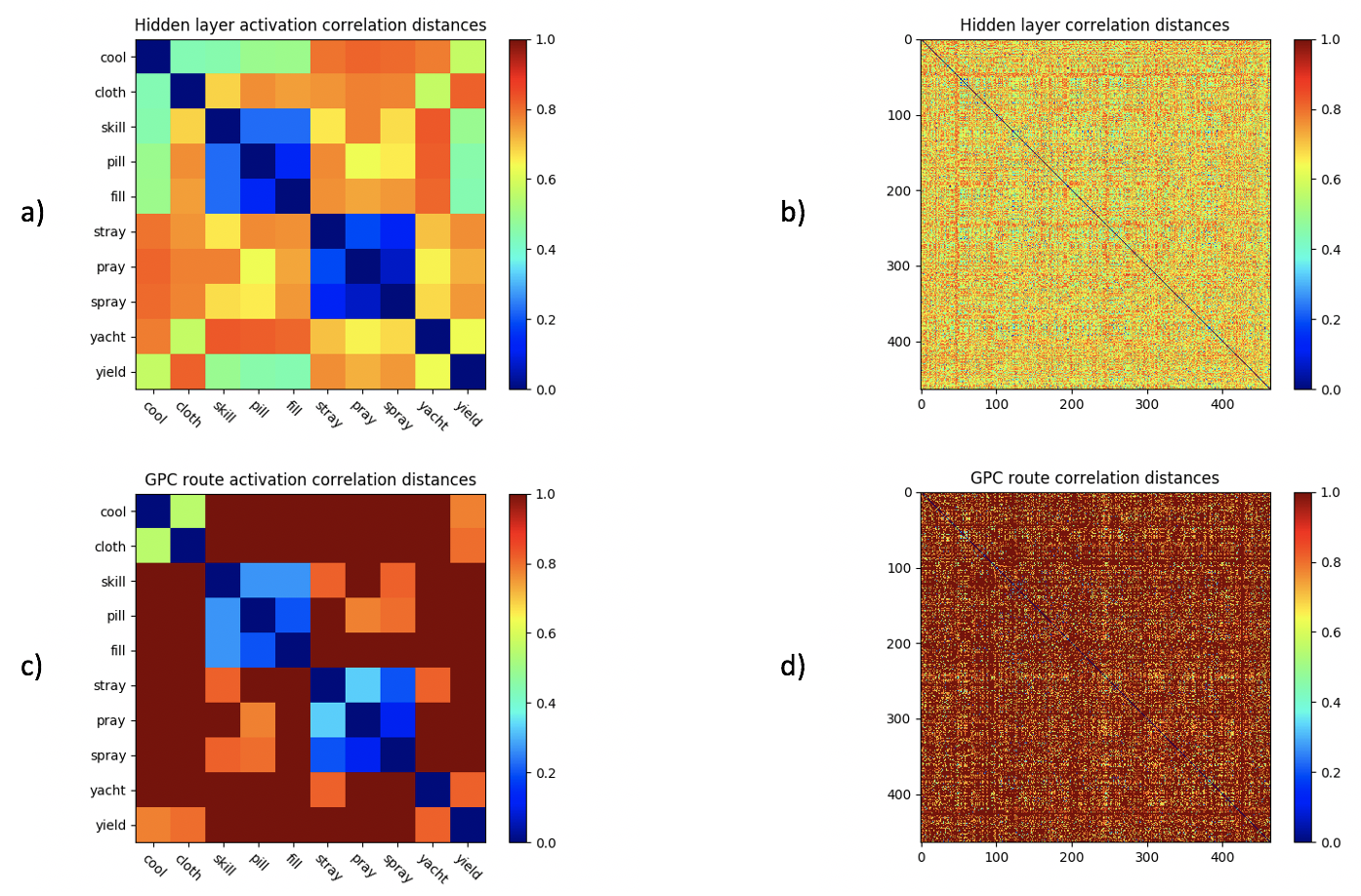


Supplementary Figure 2: a) Example hidden layer dissimilarity matrix (DSM). b) Hidden layer DSM for the 464 tested words. c) Example dual-route cascaded model grapheme-phoneme correspondence (GPC) DSM. d) GPC route DSM for the 464 tested words. See Supplementary Figure 1 for rationale of example word choice.


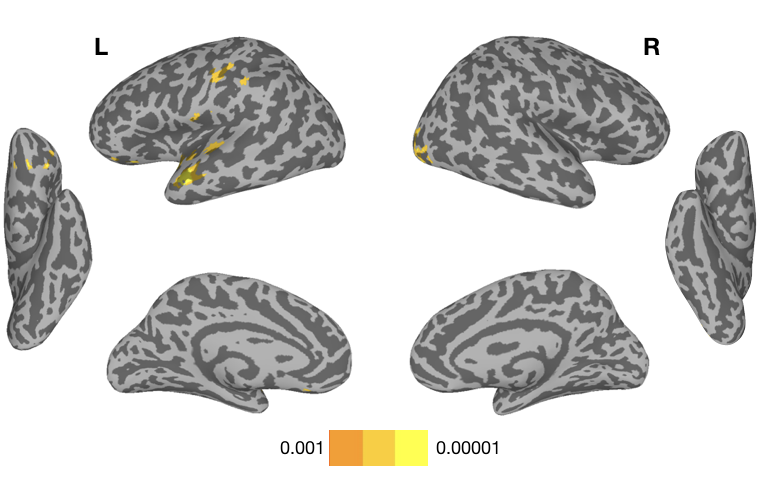


Supplementary Figure 3: Correspondence between the combined lexical and GPC route similarity structure and neural similarity structure.

|  | ANN | DRC | Orthography | Phonology |
| --- | --- | --- | --- | --- |
| ANN | 1 |  |  |  |
| DRC | 0.51 | 1 |  |  |
| Orthography | 0.9 | 0.54 | 1 |  |
| Phonology | 0.68 | 0.59 | 0.63 | 1 |

Supplementary Table 1: Correlation matrix of the dissimilarity matrices used for Representational Similarity Analysis. ANN = artificial neural network, DRC = dual-route cascaded model (GPC + Lexical representations).

| Location | Cluster Size | *x* | *y* | *z* | Z-score |
| --- | --- | --- | --- | --- | --- |
| L Inferior temporal gyrus | 218 | -37 | -7 | -37 | 4.31 |
| L Temporal pole | 140 | -22 | 11 | -28 | 3.88 |
| L Precentral sulcus | 55 | -40 | 5 | 32 | 4.18 |
| L Collateral Sulcus | 15 | -16 | -82 | -16 | 4.12 |
| L Cuneus | 15 | 2 | -94 | 11 | 3.55 |
| R Cerebellar tonsil | 11 | 29 | -28 | -37 | -13 |
| L Calcarine Fissure | 10 | -19 | -85 | -4 | 3.82 |

Supplementary Table 2: Table of orthographic input RSA cluster peaks.

| Location | Cluster Size | *x* | *y* | *z* | Z-score |
| --- | --- | --- | --- | --- | --- |
| L Superior temporal gyrus | 718 | -40 | -1 | -10 | 5.07 |
| R Inferior temporal gyrus | 124 | 47 | -10 | -31 | 4.14 |
| L Postcentral gyrus | 83 | -46 | -16 | 38 | 3.98 |
| L Middle temporal gyrus | 33 | -67 | -31 | -1 | 3.75 |
| R Inferior temporal gyrus | 31 | 53 | 8 | -34 | 4.08 |
| L Inferior parietal sulcus | 20 | -19 | -73 | 26 | 3.72 |
| L Lateral temporo-occipital sulcus | 17 | -37 | -19 | -16 | 3.79 |
| L Postcentral gyrus | 12 | -61 | -10 | 17 | 3.49 |
| L Transverse occipital sulcus | 11 | -22 | -82 | 8 | 3.45 |

Supplementary Table 3: Table of phonological output RSA cluster peaks.

| Location | Cluster Size | *x* | *y* | *z* | Z-score |
| --- | --- | --- | --- | --- | --- |
| L Middle temporal gyrus | 242 | -52 | 7 | -24 | 4.32 |
| L Inferior frontal gyrus pars orbitalis | 112 | -47 | 41 | -1 | 4.04 |
| L Precentral sulcus | 76 | -41 | 2 | 33 | 3.99 |
| R Parahippocampal gyrus | 46 | 20 | -5 | -22 | 4.41 |
| L Fusiform gyrus | 41 | -35 | -29 | -22 | 4.38 |
| L Middle orbital gyrus | 24 | -20 | 17 | -22 | 3.70 |
| L Sylvian fissure | 20 | -44 | 32 | 3 | 3.74 |
| L Inferior frontal gyrus pars triangularis | 18 | -50 | 17 | 9 | 3.51 |
| L Intraparietal sulcus | 17 | -32 | -62 | 36 | 3.82 |
| L Cuneus | 13 | -2 | -95 | 12 | 3.51 |
| L Middle temporal gyrus | 11 | -62 | -32 | -1 | 3.51 |

Supplementary Table 4: Table of ANN hidden layer RSA cluster peaks.

| Location | Cluster Size | *x* | *y* | *z* | Z-score |
| --- | --- | --- | --- | --- | --- |
| R Inferior occipital gyrus | 65 | 44 | -80 | -4 | 3.81 |
| L Anterior superior temporal sulcus | 54 | -44 | -5 | -13 | 4.30 |
| L Superior temporal gyrus | 36 | -62 | -14 | 9 | 3.63 |
| L Postcentral gyrus | 27 | -53 | -20 | 42 | 3.47 |
| L Inferior frontal gyrus pars orbitalis | 20 | -32 | 29 | -13 | 3.76 |
| L Straight gyrus | 12 | -11 | 17 | -19 | 3.77 |

Supplementary Table 5: Table of DRC grapheme-phoneme correspondence route RSA cluster peaks.

| Location | Cluster Size | *x* | *y* | *z* | Z-score |
| --- | --- | --- | --- | --- | --- |
| L Middle frontal gyrus | 27 | -44 | 53 | 2 | 3.87 |
| L Inferior frontal gyrus pars orbitalis | 18 | -47 | 38 | -1 | 3.92 |
| L Inferior temporal gyrus | 11 | -35 | -11 | -37 | 3.42 |
| R Parahippocampal gyrus | 11 | 23 | -8 | -19 | 3.67 |

Supplementary Table 6: Table of ANN-DRC partial correlation RSA cluster peaks.
